# *In Vivo* Assessment of Arsenic Bioavailability in Rice and Its Significance for Human Health Risk Assessment

**DOI:** 10.1289/ehp.9322

**Published:** 2006-08-03

**Authors:** Albert L. Juhasz, Euan Smith, John Weber, Matthew Rees, Allan Rofe, Tim Kuchel, Lloyd Sansom, Ravi Naidu

**Affiliations:** 1 Centre for Environmental Risk Assessment and Remediation, Division of Information Technology, Engineering and the Environment, University of South Australia, Adelaide, South Australia, Australia; 2 Institute of Medical and Veterinary Science, Adelaide, South Australia, Australia; 3 School of Pharmacy and Medical Sciences, Division of Health Sciences, University of South Australia, Adelaide, South Australia, Australia

**Keywords:** arsenic, arsenic daily intake values, bioavailability, *in vivo*, maximum tolerable daily intake, rice, risk assessment, speciation

## Abstract

**Background:**

Millions of people worldwide consume arsenic-contaminated rice; however, little is known about the uptake and bioavailability of arsenic species after arsenic-contaminated rice ingestion.

**Objectives:**

In this study, we assessed arsenic speciation in greenhouse-grown and supermarket-bought rice, and determined arsenic bioavailability in cooked rice using an *in vivo* swine model.

**Results:**

In supermarket-bought rice, arsenic was present entirely in the inorganic form compared to greenhouse-grown rice (using irrigation water contaminated with sodium arsenate), where most (~ 86%) arsenic was present as dimethylarsinic acid (organic arsenic). Because of the low absolute bioavailability of dimethylarsinic acid and the high proportion of dimethylarsinic acid in greenhouse-grown rice, only 33 ± 3% (mean ± SD) of the total rice-bound arsenic was bioavailable. Conversely, in supermarket-bought rice cooked in water contaminated with sodium arsenate, arsenic was present entirely in the inorganic form, and bioavailability was high (89 ± 9%).

**Conclusions:**

These results indicate that arsenic bioavailability in rice is highly dependent on arsenic speciation, which in turn can vary depending on rice cultivar, arsenic in irrigation water, and the presence and nature of arsenic speciation in cooking water. Arsenic speciation and bioavailability are therefore critical parameters for reducing uncertainties when estimating exposure from the consumption of rice grown and cooked using arsenic-contaminated water.

Arsenic contamination of groundwater has been reported in many countries throughout the world, most notably in Southeast Asia. In recent years, much attention has focused on the As calamity in Bangladesh and West Bengal, India, following the highly publicized reports of vast populations being exposed to As-contaminated groundwater. Recently, Chakraborti et al. (2004) reported that As levels in groundwater from 50 districts in Bangladesh (representing ~ 2,000 villages) exceeded the Bangladesh drinking water guidelines for As of 50 μ with As concentrations in some cases > 1,500 μ(Tondel et al. 1999). The issue in Bangladesh has been described as “the largest poisoning of a population in history” (Smith et al. 2000), with an estimated 35–70 million inhabitants being at risk of drinking As-contaminated water (Khan et al. 1997). Chronic exposure to As causes significant human health effects including various cancers (skin, lungs, bladder, and kidneys), skin disorders (hyperkeratosis and pigment changes), vascular disease, and diabetes mellitus (Guha Mazumder et al. 1998; Guo et al. 1997; Lein et al. 2001; Mandal and Suzuki 2002; Rahman et al. 2001). The increased incidence of these health effects have been observed in Bangladesh and West Bengal populations exposed to As (Ahmed et al. 2006; Mukherjee et al. 2006).

In addition to drinking water, consumption of As-contaminated food is another major source of As exposure. In Bangladesh, As-contaminated water is also used for irrigating crops, particularly rice (*Oryza sativa* L.), which represents approximately 83% of the total irrigated area in Bangladesh (Dey et al. 1996). As a consequence of irrigating with As-contaminated water, rice may contain elevated levels of As. Arsenic concentrations ranging from 160 to 580 μ have been reported in rice from the Jessore district in Bangladesh (Alam et al. 2002), whereas Meharg and Rahman (2003) reported As concentrations > 1,830 μg/kg in rice from other regions of Bangladesh. Because rice is a staple food in Bangladesh, providing > 70% of the daily calorific intake (Ninno and Dorosh 2001), consumption of contaminated rice may represent a significant As exposure pathway. In fact, Meharg (2004) estimated that consumption of As-contaminated rice may contribute as much as 60% of the daily Bangladeshi dietary As intake based on conservative As concentrations in rice. In addition, absorption of As-contaminated water during the rice cooking process may significantly increase the amount of As in cooked rice (Ackerman et al. 2005; Bae et al. 2002), which is often overlooked when calculating As daily intake values.

A number of studies have reported the presence of As in rice, ranging in concentration from 32 to 1,830 μg As/kg (Abedin et al. 2002a, 2002b; Alam et al. 2002; D’Amato et al. 2004; Heitkemper et al. 2001; Kohlmeyer et al. 2003; Meharg 2004; Meharg and Rahman 2003; Schoof et al. 1998; Williams et al. 2005); however, few studies have determined the proportion of inorganic to organic As in rice. In terms of human health risk assessment, As speciation is important because the toxicity of organic, inorganic, trivalent, and pentavalent As species vary greatly (Petrick et al. 2000; Vahter and Concha 2001). In addition, there is a dearth of information on the bioavailability of As in rice after consumption. Bioavailability, in the context of human health risk assessment, refers to the fraction of an administered dose that reaches the central (blood) compartment from the gastrointestinal tract (Ruby et al. 1999). After consumption of As-contaminated rice, it is unclear what proportion of the rice-bound As is absorbed and whether As speciation influences absorption from the gastrointestinal tract. To reduce the uncertainties in estimating exposure and to provide a more accurate estimate of risk, assessment of As bioavailability is critical.

In this study, we investigated the concentration and speciation of As in supermarket-bought rice, in rice grown under greenhouse conditions using As-contaminated irrigation water, and in rice cooked in As-contaminated water. In addition, we assessed the bioavailability of As in rice using an *in vivo* swine assay—an animal model used to predict As uptake for human health risk assessment. We assessed As bioavailability in two different rice preparations to determine whether the mode of As accumulation in the grain (translocation by the plant vs. absorption during cooking) influenced As uptake in the swine model.

## Materials and Methods

### Rice varieties, cultivation, and preparation

We used three rice varieties in this study. Supermarket-bought rice, including Basmati White (India) and Long White (Australia) rice, were purchased from a local supermarket (Adelaide, Australia), whereas Paddy rice (*Oryza sativa* Quest) was grown under greenhouse conditions.

Quest was cultivated under paddy conditions in pools containing washed sand (pH 7.5) mixed with a slow-release fertilizer low in phosphate. The slow-release fertilizer was applied at a rate consistent with nitrogen and potassium rates applied in field conditions (70 kg/ha). Rice seeds were germinated in moist compost and planted into pools 3 weeks after germination. After transplantation, each pool contained 75 seedlings which were exposed to a 16-hr light period with the temperature maintained at 28 ± 5°C. Plants were grown to maturity (30 weeks) under paddy field conditions (i.e., saturation with 30–40 mm standing water) with irrigation water containing 1,500 ± 300 μg As/L supplied as Na_2_HAsO_4_·7H_2_O (arsenate; As^V^). This As concentration was selected because it represented the highest concentration of As reported in contaminated groundwater in Bangladesh (Tondel et al. 1999). No additional fertilizer supplements were added to the rice during the growing period. On maturity, rice heads were harvested and air dried for 10 days. Rice heads were manually threshed using a stainless steel thresher frame and a polyethylene grooved board. When cooked rice was required, rice was prepared using the absorption method. Quest was cooked in As-free water (1:2.5 v/v rice to water), whereas Basmati White was cooked in water (1:2.5 v/v rice to water) containing 1,000 μg As/L supplied as As^V^.

### Determination of As concentration in rice

We analyzed Basmati White, Long White, and Quest for total As concentration by digesting approximately 0.5 g rice with concentrated HNO_3_ (10 mL). Digestion tubes were allowed to stand overnight at room temperature; the following day, the tubes were placed on a heating block and the temperature increased in steps from 75 to 140°C for up to 10 hr. Digested samples were removed from the heating block when nitric acid volumes were reduced to 1 mL. Once the digests had cooled, samples were diluted to 20 mL with deionized water and filtered (0.45-μm filters) before analysis by inductively coupled plasma–mass spectrophotometry (ICP-MS; Agilent Technologies, Melbourne, Australia). For quality assurance and quality control, the appropriate number of blank and standard reference material samples [certified reference material (CRM) DC73349; Rowe Scientific, Perth, Western Australia, Australia] were included in the digestion procedure and sample analysis.

### Speciation of As in the rice grain

The nature of As speciation in rice grains was determined using the trifluoroacetic acid (TFA) extraction technique of Abedin et al. (2002b). After grinding of rice in a stainless steel grinder (Breville, Sydney, New South Wales, Australia), a portion (0.25 g) of rice material was weighed into 100-mL glass digestion tubes to which 2 mL 2 M TFA was added. The digestion tubes were placed on a heating block, and the temperature was increased to 100°C for 6 hr. The digest was evaporated to dryness and the residue dissolved in deionized water, filtered (0.22-μm filters), and made up to 20 mL with deionized water. The extracts were stored at −20°C before analysis by high performance liquid chromatography (HPLC)-ICP-MS (Agilent Technologies).

The nature and concentration of As species in extract solutions was determined by HPLC-ICP-MS (Agilent Technologies). Samples were injected onto a PRP-X100 anion-exchange column (250 × 4.1 mm internal diameter, 10 μm; Hamilton, Reno, NV, USA) using a fixed 50-μL sample loop. The column temperature was maintained at 40°C and the mobile phase (20 mM NH_4_H_2_PO_4_ adjusted to pH 5.6 with aqueous NH_3_) flow rate was 1.5 mL/min. We quantified As compounds by external calibration with standard solutions of arsenite (As^III^), As^V^, dimethylarsinic acid (DMA), and monomethylarsonic acid (MMA) (Akter et al. 2005).

### Assessment of As bioavailability—in vivo assays

*In vivo* assays were approved and conducted according to application 1702 of the Institute for Medical and Veterinary Science Animal Ethics Committee. Animals used in the study were treated humanely and with regard for the alleviation of suffering. Female Large White swine, weighing 20–25 kg, were used for *in vivo* bioavailability assays. After acclimation for 12 days to animal house conditions, swine were fasted for 24 hr before surgery for the insertion of jugular catheters. We used medical-grade vinyl tubing (550-mm lengths; Microtube Extrusions, North Rocks, New South Wales, Australia) for all catheterizations, according to the method of Bain et al. (1991) with minor modification. Adjustable electrical clips (3 mm; Farnell InOne, Chester Hill, New South Wales, Australia) with fixing holes were fitted over the catheter and held firmly with small electrical zip ties, to grip the catheter. Clips were sutured to muscle tissue surrounding the cannulated external jugular to stabilize the catheter *in situ*. A 15-gauge blunt luer needle fitting (Monoject; Sherwood Medical, St. Louis, MO, USA) was fitted to catheter ends. Interlink injection site bungs (Baxter Healthcare Corp., Deerfield, IL, USA) were attached when catheters were not in use. Extension tubing (1,500 mm, Minimum Volume Extension Set; Tuta Healthcare, Lane Cove, New South Wales, Australia) connected to a three-way tap (Connecta Plus 3; Becton Dickinson, Franklin Lakes, NJ, USA) was fitted to catheter ends when sampling blood during experiments.

During bioavailability assays, animals were housed in metabolic cages. Swine were fed twice daily [500 g low-As swine pellets (10 ± 5 μg As/kg)], 2 and 10 hr after As dosage while water was supplied *ad libitum*. Before As dosage, blood samples were taken to determine baseline blood As concentrations. Catheters were then flushed with 20 mL heparinized saline (50 IU Heparin/mL). For oral As dosage, solutions of MMA, DMA, As^III^, or As^V^ (80–100 μg As/kg) were supplied in 150 mL deionized water after intravenous administration of diazepam (2,000 μg/kg) and ketamine (5,000 μg kg^−1^) to induce short-term anesthesia to facilitate the passing of a gastric tube. Intravenous As dosages (MMA, DMA, As^III^, As^V^: 20 μg As/kg) were administered using a catheter separate from the blood sampling catheter. Arsenic-contaminated rice (170–270 g) was fed to animals with 20 g pelletized food to increase palatability. Blood samples were routinely taken over 26 hr after dosage and collected in 7.5-mL heparinized collection tubes (S-Monovette 7.5 mL LH-Gel; Sarstedt, Rommelsdorfer Strabe, Germany). After each blood sample, catheters were flushed with saline. Catheters were flushed with 20 mL heparinized saline after 6-, 10-, 24-, and 26-hr samples. Plasma was separated from red blood cells by centrifugation (4,000 rpm for 10 min) and then stored at −20°C before As analysis. For each *in vivo* treatment (intravenous dose, oral dose, or rice dose), three separate animals were used. The concentration of As in blood plasma was determined by ICP-MS (Agilent Technologies) and As bioavailability calculated using pharmacokinetic analysis encompassing areas under the plasma-concentration [area under the curve (AUC)] time curves after zero correction and dose normalization. When calculating the absolute bioavailability of As species, the AUC for the respective As intravenous treatment was used and compared to oral doses (Equation 1).


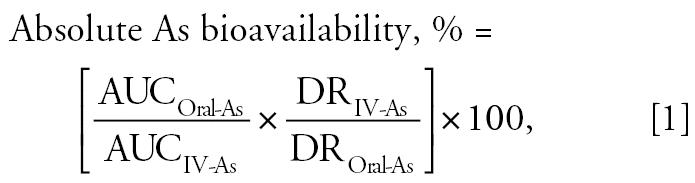


where AUC_Oral-As_ is area under the As blood plasma concentration versus time curve for an oral arsenic dose; AUC_IV-As_ is area under the As blood plasma concentration versus time curve for an intravenous arsenic dose; DR_IV-As_ is dose of intravenously administered arsenic (milligrams per kilogram); and DR_Oral-As_ is dose of orally administered arsenic (milligrams per kilogram).

When calculating the absolute bioavail-ability of As in rice, we compared As speciation data and AUC values for rice doses to intravenous As treatment:


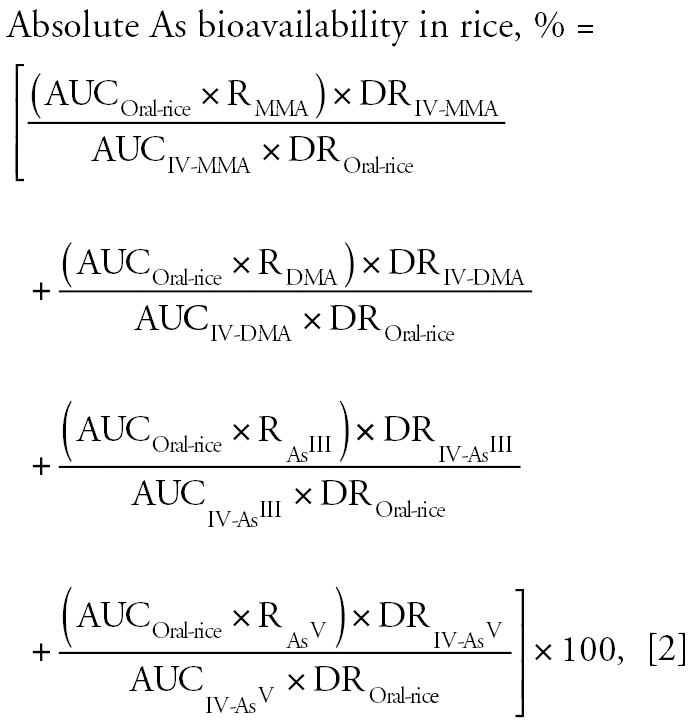


where AUC_Oral-rice_ is area under the As blood plasma concentration versus time curve for an oral rice dose; AUC_IV_ is area under the As blood plasma concentration versus time curve for an intravenous arsenic dose (MMA, DMA, As^III^, or As^V^); R is ratio (fraction of 1) of either As species (MMA, DMA, As^III^, or As^V^) in the rice; DR_IV_ is dose of intravenously administered arsenic (MMA, DMA, As^III^, or As^V^) (milligrams per kilogram); and DR_Oral-rice_ is dose of orally administered As in rice (milligrams per kilogram)

### Determination of As in blood plasma

We used two methods to determine As in blood plasma. Samples (3 mL) were digested with nitric acid (2 mL; 70%) and H_2_O_2_ (1 mL; 30%) using U.S. Environmental Protection Agency method 3015A (U.S. Environmental Protection Agency 1998). After digestion, samples were diluted with Milli-Q water to reduce the acid content to 10%. Alternatively, blood serum was diluted 10-fold in diluent solution containing 1-butanol (2% w/v), EDTA (0.05% w/v), Triton X-100 (0.05% w/v), and ammonium hydroxide (1% w/v) in Milli-Q water (Agilent Technologies 2006) before analysis. All samples were analyzed by ICP-MS (Agilent Technologies) with the appropriate number of duplicate samples, duplicate analysis, spiked sample recoveries, and check values included for quality assurance and quality control.

## Results and Discussion

### Quality assurance and quality control

During the analysis of total As concentration in rice samples, a standard reference material (CRM DC73349) was included in the digest and analytical procedures for quality assurance and quality control. The accuracy of the HNO_3_ digestion method was confirmed by a quantitative average As recovery of 30.05 ± 0.87 mg/kg (*n* = 4) from CRM DC73349 samples (26.18 ± 3.14 mg As/kg). During the determination of total As concentration in rice and plasma samples, duplicate analysis, spiked sample recoveries, and check values were included. The average deviation between duplicate samples (*n* = 16) was 3.8% (0.2–8.5%), the average recovery from spiked samples (*n* = 8) was 103% (101–109%), whereas check value recoveries (*n* = 32) ranged from 94.2 to 106.7% (101.5% average recovery). In addition, we assessed the accuracy of the As speciation method by analyzing As standard solutions (MMA, DMA, As^III^, and As^V^; 100 μg/L) during the speciation procedure. Recoveries for MMA, DMA, As^III^, and As^V^ were 92 ± 3% (*n* = 6).

### As concentration and speciation in rice

Several studies (Abedin et al. 2002a, 2002b; Bae et al. 2002; Meharg 2004; Meharg and Rahman 2003) have determined the total concentration of As in rice from various regions around the world; however, few studies have investigated the speciation of As in rice grains. Table 1 illustrates the variability in As concentration and speciation in cooked and uncooked rice from market surveys and greenhouse experiments. We performed As analysis on three rice varieties, of which two were bought from a local supermarket (Basmati White and Long White), whereas the other (Quest) was grown under greenhouse conditions, irrigated with As-contaminated water. The Basmati White rice variety, produced in India, contained low concentrations of As (32 ± 3 μg/kg), whereas the Australian Long White variety contained 189 ± 11 μg As/kg. In greenhouse-grown rice, however, Quest accumulated 1,250 ± 230 μg As/kg in the grain, which was significantly higher than concentrations reported in previous studies using greenhouse-grown and market-bought rice (Table 1). In these studies, the As concentration in rice ranged from 70 to 760 μg As/kg for market-bought rice (D’Amato et al. 2004; Heitkemper et al. 2001; Kohlmeyer et al. 2003; Schoof et al. 1998; Williams et al. 2005). In addition, Abedin et al. (2002a) reported As concentrations ranging from 150 to 420 μg As/kg in rice grown under greenhouse conditions using As-contaminated irrigation water. Although Quest was grown using an elevated concentration of As in the irrigation water (1,500 ± 300 μg/L), the experiment demonstrates the propensity for rice to accumulate elevated concentrations of As in the grain given the appropriate environmental conditions.

When rice was cooked, the concentration of As in the grain varied depending on the concentration of As in the cooking water. Cooking Quest in uncontaminated water (MilliQ) produced a final As concentration of 480 μg/kg (wet weight) as a result of absorption of water during the cooking process. However, when Basmati White rice was cooked in water containing 1,000 μg As^V^/L, the As concentration increased from 32 to 1,000 μg/kg (Table 1). In a previous study, Ackerman et al. (2005) reported that As concentrations increased between 13 and 31% when Instant White or Long White were cooked in water containing 21.9 μg As^V^/L (Table 1). Bae et al. (2002) suggested that the increase in As concentration in rice after cooking in contaminated water resulted from a chelating effect by rice grains, or concentration of As due to water evaporation during the cooking process or both.

After the determination of As concentration in rice, daily As intake values can be easily calculated. Assuming consumption of rice at a rate representative of a rice-based subsistence diet (0.42 kg dry weight/day) (Baffes and Gautan 2001), the daily As intake from the consumption of Basmati White and Quest would be 13.4 and 525 μg As, respectively. Assuming an average body weight of 60 kg, consumption of Basmati White would represent 11% of the World Health Organization’s (WHO) provisional maximum tolerable daily intake (MTDI) of 2 μg/kg As (WHO 1993), whereas consumption of Quest would exceed the MTDI value four-fold. Another important factor is that the concentration of As in cooked rice will be exacerbated by the concentration of As in the cooking water, which will affect As daily intake calculations. Bae et al. (2002) noted an increase from 173 to 222–377 μg As/kg when rice was cooked in water containing 223 to 372 μg As/L resulting in an increase in As concentration of between 28 and 118%.

We performed As speciation on uncooked and cooked Basmati White and Quest rice to determine the proportion of organic and inorganic As in the grain. Determination of As speciation in rice is important because the toxicity of organic, inorganic, trivalent, and pentavalent As species vary greatly (Petrick et al. 2000; Vahter and Concha 2001). Arsenic was present entirely in the inorganic form in Basmati White (Table 1); however, 86 ± 2% of As in Quest was present as DMA (organic As). The remaining As (14 ± 2%) was present as As^III^. Previous studies have demonstrated the variability in As speciation in rice varieties from around the world (Table 1). In a market survey study by Williams et al. (2005), organic As in rice from Bangladesh, India, Italy, Spain, Thailand, and the United States represented 6–65% of the total rice As content. Similarly, the proportion of organic As in rice studies conducted by Ackerman et al. (2005), D’Amato et al. (2004), Heitkemper et al. (2001), Kohlmeyer et al. (2003), and Schoof et al. (1998) ranged from 6 to 89%. These results indicate that As speciation in rice is highly variable depending on rice cultivar, locality, and growing conditions (Williams et al. 2005).

### As bioavailability in rice

Based on consumption rates and the concentration of As in rice, daily As intake values are easily calculated. However, when determining intake values, it is assumed that 100% of the As is bioavailable (i.e., the As is absorbed and enters systemic circulation). The paucity of absorption data and the expense and difficulty in performing relevant bioavailability studies have led to a conservative approach regarding As absorption from food in human health risk assessment. In fact, reducing the uncertainties in estimating exposure of As in food through bioavailability studies was a key recommendation for future research from Environmental Health Criteria 224, *Arsenic and Arsenic Compounds* (WHO 2001). To address this shortfall in bioavailability data, we performed As bioavailability studies with Quest and Basmati White using an *in vivo* swine assay. Immature swine are the animal of choice for *in vivo* As bioavailability studies because they are similar to humans in digestive tracts, nutritional requirements, bone development, and As metabolism (Weis and LaVelle 1991).

**Figure 1:**
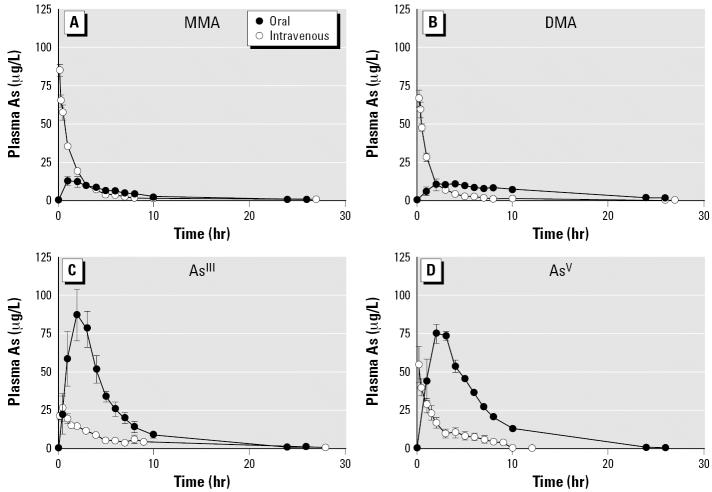
Arsenic concentration in blood plasma after oral or intravenous administration of (*A*) MMA, (*B*) DMA, (*C*) As^III^, or (*D*) As^V^. Each swine received a single administration of 20 μg As/kg or 80–100 μg As/kg for intravenous and oral doses, respectively. Data points represent the mean ± SD of three separate *in vivo* assays.

Initially, pharmacokinetic studies were performed with MMA, DMA, As^III^, and As^V^ to determine the absolute bioavailability of these As species (Figure 1). We determined absolute bioavailability by comparing areas under the plasma As concentration time curve for oral and intravenous routes of administration (Equation 1). For inorganic As, the absolute bioavailability of these species was approximately 100% although some variability was observed among animal treatments. For As^III^ and As^V^, 103.9 ± 25.8% and 92.5 ± 22.3% of the administered oral dose was absorbed from the gastrointestinal tract and entered systemic circulation respectively (Table 2). In contrast, organic arsenic was poorly absorbed after oral administration, resulting in low absolute bioavailability values. In treatments where MMA was supplied orally, only 16.7 ± 5.0% of the administered dose entered systemic circulation compared to the intravenous treatment. DMA was also poorly absorbed from the gastrointestinal tract with 33.3 ± 1.7% of the DMA oral dose entering systemic circulation (Table 2).

We determined the absolute bioavailability of As in rice after pharmacokinetic studies using two different rice treatments. Quest (1,250 ± 230 μg As/kg) was cooked in “uncontaminated water” and then fed to the swine to determine the absolute bioavailability of As in rice after cultivation using As-contaminated irrigation water. In addition, Basmati White was cooked in “As-contaminated water” (1,000 μg As^V^/L) to increase its As content (32 to 1,000 μg/kg) and then fed to the swine. This treatment was performed to determine the bioavailability of As absorbed during the cooking process. Absolute As bioavailability in these rice treatments was determined according to Equation 2. Data from speciation studies was included in bioavailability calculations because of the observed variability in gastrointestinal absorption of different As species.

Results from *in vivo* swine assays demonstrated that As bioavailability in Quest and Basmati White varied considerably. In rice grown using As-contaminated irrigation water (Quest), only 33.1 ± 3.2% of As was absorbed into systemic circulation (Table 2). The low bioavailability of As in Quest was a result of the high proportion of DMA present in the rice. Speciation studies identified DMA as the major As species present in Quest, representing 86% of the total As concentration (Table 1). DMA was shown to be poorly absorbed in pharmaco-kinetic studies after administration of an oral dose; only a third of the oral dose entered systemic circulation compared to intravenous treatments (Table 2).

In contrast, Basmati White cooked in As-contaminated water contained entirely inorganic As as a result of As^V^ supplied to the cooking water (Table 1). After consumption of basmati white, 89.4 ± 9.4% of As was absorbed into systemic circulation (Table 2). A previous study examining the bioaccessibility/ bioavailability of As in rice cooked in As-contaminated water using simulated *in vitro* gastrointestinal digestion and Caco-2 cells found that As bioaccessibility ranged from 63 to 99% (Laparra et al. 2005). However, As uptake by Caco-2 cells varied from 3.9 to 17.8% suggesting that other soluble components of the rice may limit the extent of As absorption. The low As absorption values observed by Laparra et al. (2005) may reflect the simplistic *in vitro* gastrointestinal digestion methods used, which may not reflect digestion processes occurring *in vivo*.

When calculating the contribution of rice consumption to MTDI values, the inclusion of As speciation and bioavailability data produces significantly different values compared with calculations using total rice-bound As concentrations (Table 3). In Table 3 for comparison, the contribution of rice consumption to MTDI values were calculated for a number of rice varieties from this study and the literature based on total As, inorganic As, and the bioavailability of organic and inorganic As. In calculating MTDI values, we assumed that rice was consumed by a 60-kg person at a rate of 0.42 kg dry weight/day, that DMA represented organic As, and that DMA had an absolute bioavailability of 33% (Table 2).

The most striking difference in MTDI calculations occurred for rice varieties containing high proportions of organic As (e.g., Long White, Instant White, and Quest; Table 3). Consumption of rice produced in this study (Quest) would contribute 438% of the As MTDI value based on total As concentration; however, this value would be reduced to 185% by including speciation and bioavail-ability data. As a result, intake values would be comparable to White rice (Schoof et al. 1998; see Table 3) even though the total As concentration in Quest is two-fold greater. Determination of MTDI values based solely on the total As concentration in rice may significantly overestimate As intake for varieties containing a high proportion of DMA. Conversely, inclusion of only the inorganic As concentration in MTDI calculations would underestimate As intake for varieties containing a high proportion of DMA. For Long White, Instant White, and Quest varieties, MTDI values calculated using the inorganic As concentration are 2.1–3.8 times lower than values derived using As bioavailability (Table 3).

## Conclusion

The results from this study demonstrate that As speciation plays a major role in determining the amount of As absorbed after consumption of As-contaminated rice. DMA was poorly absorbed *in vivo* after oral administration, resulting in low bioavailability values for rice containing high proportions of this As species. Conversely, As bioavailability was high in rice containing high proportions of inorganic As as a result of cooking the rice in As^V^-contaminated water. To the best of our knowledge, this is the first study that has assessed the bioavailability of As in rice using a suitable animal model for human health risk assessment. Studies of this nature, incorporating As speciation and bioavailability, are critical to reduce uncertainties in estimating exposure and to provide a more accurate estimate of risk.

## Figures and Tables

**Table 1:** Arsenic concentration and speciation in cooked and uncooked rice from market surveys and greenhouse studies (mean ± SD).

Rice variety	Source	Total As (μg As/kg)	Organic As (%)	Inorganic As (%)	As extraction efficiency (%)	Reference
Uncooked rice–market bought						
Parija	Bangladesh	210 ± 20	24 ± 0	59 ± 1	83 ± 1	Williams et al. 2005
Miniket	Bangladesh	220 ± 10	16 ± 0	86 ± 4	103 ± 4	Williams et al. 2005
BRRIdhan29	Bangladesh	300 ± 10	11 ± 2	71 ± 0	82 ± 2	Williams et al. 2005
White	Taiwan	760	14	67	81	Schoof et al. 1998
Long White	USA	400 ± 10	65 ± 1	20 ± 1	85 ± 0	Williams et al. 2005
Long Brown	USA	340 ± 20	45 ± 11	41 ± 5	86 ± 16	Williams et al. 2005
Basmati White	India	50 ± 0	23 ± 4	65 ± 1	88 ± 5	Williams et al. 2005
Basmati White	India	32 ± 3	0	100	81	This study
Basmati Brown	India	70 ± 10	6 ± 3	61 ± 4	67 ± 8	Williams et al. 2005
Medium Risotto	Italy	220 ± 10	38 ± 1	65 ± 1	103 ± 2	Williams et al. 2005
Arborio	Italy	211 ± 7	31 ± 3	66 ± 5		D’Amato et al. 2004
Paella	Spain	170 ± 10	30 ± 5	48 ± 2	78 ± 3	Williams et al. 2005
Long Jasmine	Thailand	110 ± 10	24 ± 6	74 ± 1	98 ± 7	Williams et al. 2005
Ground	Europe	200 ± 10	28 ± 2	51 ± 3	79 ± 4	Williams et al. 2005
Long Wild Rice	Canada	110	8	76	84	Heitkemper et al. 2001
Long White	Australia	189 ± 18	ND	ND	ND	This study
Not specified		410	6	88	94	Kohlmeyer et al. 2003
Uncooked rice–greenhouse grown						
Quest	Australia	1,250 ± 230	86 ± 2	14 ± 2	100 ± 4	This study
Cooked rice–market bought^a^						
Instant White	USA	305	89 ± 4	10 ± 1	99 ± 4	Ackerman et al. 2005
Instant White^b^	USA	345	78 ± 2	17 ± 1	95 ± 3	Ackerman et al. 2005
Long White	USA	236	62 ± 1	35 ± 3	97 ± 4	Ackerman et al. 2005
Long White^b^	USA	310	50 ± 3	46 ± 2	96 ± 4	Ackerman et al. 2005
Not specified		150	29	75	104	Kohlmeyer et al. 2003
Basmati White^c^	India	1,000	0	100	100	This study
Cooked rice–greenhouse grown						
Quest	Australia	480	86 ± 2	14 ± 2	100 ± 4	This study

ND, not determined.

a Cooked rice prepared with water:rice ratios of 1:1 to 4:1 (v/v) depending on variety.

b Rice cooked with water containing 21.9 μg As^V^/L.

c Rice cooked with water containing 1,000 μg As^V^/L.

**Table 2:** Absolute bioavailability of organic, inorganic, and rice-bound arsenic after *in vivo* assessment using the swine animal model (*n* = 3).

Treatment	Dose (μg As/kg)	AUC^a^	Absolute bioavailability (%)^b^
Intravenous			
MMA	20	122.7 ± 13.6	100
DMA	20	84.9 ± 3.6	100
As^III^	20	87.6 ± 14.6	100
As^V^	20	115.2 ± 40.6	100
Oral gavage			
MMA	100	92.8 ± 26.3	16.7 ± 5.0
DMA	100	138.2 ± 1.1	33.3 ± 1.7
As^III^	80	483.7 ± 172.9	103.9 ± 25.8
As^V^	100	463.8 ± 45.7	92.5 ± 22.3
Rice			
Quest^c^	3.3–5.2	3.6–6.6	33.1 ± 3.2^d^
Basmati^e^	16.5–20.2	71.8–87.2	89.4 ± 9.4^d^

a Area under the curve data represents the mean ± SD of triplicate analyses.

b Absolute bioavailability was calculated using the Equation 1 (see “Materials and Methods”).

c As-contaminated rice was cooked in As-free water.

d The bioavail-ability of As in Quest and Basmati White rice was calculated using speciation data outlined in Table 1 using Equation 2 (see “Materials and Methods”).

e Supermarket-bought rice was cooked in water containing 1,000 μg As^V^/L.

**Table 3:** Contribution of rice consumption to maximum tolerable daily intake calculations using total rice-bound As, inorganic As, and bioavailable As.

				Contribution to MTDI (%) based on:		
Rice Variety	Total As (μg/kg)	Organic As^a^ (μg/kg)	Inorganic As^a^ (μg/kg)	Total As^b^	Inorganic As^c^	As bioavailability^d^
Parija^e^	210	50	124	74	43	49
Miniket^e^	220	35	189	77	66	70
BRRIdhan29^e^	300	33	213	105	75	78
White^f^	760	106	509	266	178	190
Long White^e^	400	260	80	140	28	58
Long Brown^e^	340	153	139	119	49	66
Basmati White^e^	50	12	33	18	12	13
Basmati White^g^	32	0	32	11	11	11
Basmati Brown^e^	70	4	43	25	15	16
Medium Risotto^e^	220	84	143	77	50	60
Arborio^h^	210	65	139	74	49	56
Paella^e^	170	51	82	60	29	35
Long Jasmine^e^	110	26	81	39	28	31
Ground^e^	200	56	102	70	36	42
Long Wild^i^	110	9	84	39	29	30
Not specified^j^	410	25	361	144	126	129
Quest^g^	1,250	1,075	175	438	61	185
Instant White^k^	305	271	31	107	11	42
Long White^k^	236	146	83	83	29	46
Not specified^j^	150	44	113	53	40	45

a Organic and inorganic As concentrations were calculated from percentage values presented in Table 1.

b The contribution to MTDI As values (%) was calculated using consumption of 0.42 g rice dry weight/day for a 60-kg person and the total As concentration for each rice variety.

c The contribution to MTDI As values (%) was calculated using consumption of 0.42 g rice dry weight/day for a 60-kg person and the concentration of inorganic As for each rice variety.

d The contribution to MTDI As values (%) was calculated using consumption of 0.42 g rice dry weight/day for a 60-kg person, the concentration of organic and inorganic As and bioavailability factors of 0.33 and 1.0 for organic and inorganic As respectively. DMA was assumed to represent the organic As fraction.

e Williams et al. 2005.

f Schoof et al. 1998.

g This study.

h D’Amato et al. 2004.

i Heitkemper et al. 2001.

j Kohlmeyer et al. 2003.

k Ackerman et al. 2005.
